# Development and use of a toolkit to facilitate implementation of an evidence-based intervention: a descriptive case study

**DOI:** 10.1186/s43058-020-00081-x

**Published:** 2020-10-06

**Authors:** Kelli Thoele, Melora Ferren, Laura Moffat, Alyson Keen, Robin Newhouse

**Affiliations:** 1 Robert Wood Johnson Future of Nursing Scholar, Indiana University School of Nursing, 600 Barnhill Drive, Indianapolis, IN 46202 USA; 2 Indiana University School of Nursing, 600 Barnhill Drive, Indianapolis, 46202 IN USA; 3 Indiana University Health, Fairbanks Hall, 340 W. 10th Street, Indianapolis, IN 46202 USA; 4 Indiana University Health Arnett Hospital, 5165 McCarty Lane, Lafayette, IN 47905 USA; 5 Indiana University Health Adult Academic Health Center, 1701 N. Senate Blvd, Indianapolis, IN 46202 USA

**Keywords:** Implementation toolkit, Evidence-based practice, Implementation strategies, Acute care, SBIRT, Nurse, Case report

## Abstract

**Background:**

Implementation of evidence-based clinical interventions in real-world settings becomes a futile effort when effective strategies to foster adoption are not used. A toolkit, or a collection of adaptable documents to inform and facilitate implementation, can increase the use of evidence-based interventions. Most available toolkits provide resources about the intervention but lack guidance for adaptation to different contexts or strategies to support implementation. This paper describes the development and use of a toolkit to guide the implementation of an evidence-based intervention to identify and intervene for people with risky substance use.

**Methods:**

A descriptive case study describes the development and use of a toolkit throughout a two-year study. Investigators and site coordinators from 14 acute care hospitals developed tools and engaged external stakeholders as they prepared for implementation, integrated the clinical intervention into practice, and reflected on implementation.

**Results:**

The final toolkit included 54 different tools selected or created to define the intervention, engage and communicate with stakeholders, assess for readiness and plan for implementation, train clinical nurses and other stakeholders, evaluate training and implementation effectiveness, create policies and procedures for different contexts, and identify opportunities for reimbursement. Each tool corresponds to one or more implementation strategies.

**Conclusion:**

The approach used to develop this implementation toolkit may be used to create resources for the implementation of other evidence-based interventions.

Contributions to the literature

- Toolkits can be used to facilitate the integration of evidence-based interventions into practice.
- Several toolkits exist to facilitate the implementation of evidence-based interventions; however, attention to implementation strategies and context is often lacking.
- This article contributes to the literature regarding the development and use of toolkits with input from diverse stakeholders to facilitate implementation in various contexts via multiple implementation strategies.

## Background

Evidence-based clinical interventions in healthcare are associated with increased quality of care, improved patient outcomes, and reduced healthcare costs [1]. Despite these positive outcomes, healthcare providers report evidence-based interventions are ineffectively implemented in real-world settings [2–6]. Healthcare providers have favorable attitudes about evidence-based practice; however, their intention to adopt an intervention does not consistently translate to the actual implementation of the intervention [7, 8]. This gap between evidence and practice is potentially detrimental and may result in preventable morbidity and mortality for healthcare recipients [9–11].

To address this gap between evidence and practice, Powell and colleagues have identified several strategies to support the implementation of evidence-based interventions, including identification of champions, assessment for adoption readiness, identification of barriers, and promotion of the adaptability of the intervention [12]. Healthcare providers and leaders may develop tools (i.e., documents that provide information or guidance) that align with strategies used to support implementation. For example, a “Capacity Assessment Tool” may provide a framework by which leaders assess readiness for implementation. A collection of tools, or a toolkit, may include adaptable resources that expedite the translation of evidence into practice with a specific focus on a single intervention or audience. Toolkits may include documents which may be used individually or collectively, such as educational material, timelines, agenda templates, and assessment tools [13], and the tools may often be customized based on context [14], thus helping to bridge the translation gap between evidence and practice. Additionally, these tools may be used throughout the implementation process to support adoption (the decision to use the intervention), implementation (incorporation of the intervention into practice), and sustainment (continued use of the intervention).

The use of a toolkit to support the implementation of a clinical intervention is associated with improved patient outcomes, including reduced falls [15], reduced number of hyperglycemic events, and reduced length of stay [16]. Development of a toolkit from start to finish is a multi-step process, and methods for toolkit development may include interviewing healthcare providers [16–19], conducting observations or site visits [18], and using a Delphi approach [18, 20]. Toolkits to support the implementation of evidence-based interventions commonly target healthcare providers, although toolkits may also inform community partners, patients, and other stakeholders [21, 22]. Although toolkits are associated with improved clinical outcomes [22], the content of toolkits often focus on the steps required to complete the clinical intervention with less emphasis on the strategies used to facilitate implementation in real-world settings. Additionally, toolkits often include resources to provide education, and tools to assess implementation fidelity and implementation outcomes are less-commonly included [22].

The purpose of this article is to describe the development and use of a toolkit to facilitate the implementation of an evidence-based clinical intervention. This descriptive case study focuses on the creation of a toolkit for healthcare workers, in the acute care setting, who deliver a clinical intervention to patients with a substance use disorder. The toolkit provides the blueprint to guide both the clinical intervention and the implementation activities. Although this article describes the development and use of a toolkit for substance use disorders, the structure of the toolkit and process for toolkit development described in this article may be of interest to healthcare leaders and clinicians implementing any evidence-based clinical intervention in healthcare settings.

A clinical intervention referred to as Screening, Brief Intervention, and Referral to Treatment (SBIRT) demonstrates effectiveness in the identification, treatment, and prevention of substance use disorders [23]. SBIRT implementation is associated with reductions in healthcare costs, traumatic injuries, and the severity of drug and alcohol use [24]. SBIRT is an evidence-based intervention; yet, global adoption by healthcare professionals has been limited, with the use of SBIRT noted more often in the primary care and emergency department settings [25–29]. Patients presenting to the acute care setting with substance use disorder represent a large population that is not commonly receiving SBIRT interventions.

Approximately 15% of hospitalized adults have a substance use disorder [30]. Substance abuse in the Midwest region of the USA reflects national trends; however, this region has its own set of challenges, including increasing rates of overdose fatalities [31]. At a large healthcare system in the Midwest, nurse leaders found that healthcare providers did not routinely screen hospitalized patients for substance use. In addition, there was not a standard process for intervening or providing a referral to treatment. Although there are SBIRT toolkits publically available [32, 33], these toolkits primarily focus on the clinical intervention (SBIRT) with less emphasis on the creation of tools to support the implementation of SBIRT in various settings.

Stakeholders from the Indiana University School of Nursing and Indiana University Health healthcare system developed an interdisciplinary team of investigators to evaluate the use of a toolkit to facilitate the implementation of SBIRT in acute care. The parent study, a phased cluster-randomized controlled study, included a train-the-trainer approach with site coordinators leading and championing implementation at each facility [34]. The aims of the parent study were to (1) test if implementation of SBIRT led to an increase in the percentage of patients who received SBIRT and (2) evaluate the cost of SBIRT implementation [34]. While investigators for the parent study developed an initial toolkit, this toolkit was modified and refined throughout the implementation process. This article will describe the development and use of the implementation toolkit in three phases: (1) preparation and site coordinator training, (2) readiness assessment and implementation, and (3) reflection and refinement of the toolkit. This multi-step process included the selection and application of existing tools, the iterative development of new tools throughout the implementation process, and the post-implementation refinement of tools.

## Methods

### Study design and setting

This descriptive case report describes the process of developing a toolkit while implementing SBIRT in acute care hospitals from August 2017 through June 2019. The parent study [34] was conducted at 14 acute care hospitals within one healthcare system in the Midwest region of the USA. Indiana University Health is the largest healthcare system in Indiana, and all of the non-pediatric hospitals at Indiana University Health participated in the parent study. The participating hospitals included non-profit teaching and non-teaching hospitals in urban, suburban, and rural settings; Hospital bed size ranged from 15 to 858 beds. The CARE case report guidelines were used to inform the reporting of this case study.

### Development and use of the toolkit

To create the toolkit, investigators reviewed several resources for toolkit development and selected the resource described by the University of California Berkeley School of Social Welfare (CalSWEC) [35]. Investigators selected the CalSWEC resource because this approach includes multiple considerations for successful implementation including the use of champions and networking; support from multiple stakeholders; intervention fidelity as well as the ability to adapt the intervention; and the use of multiple strategies to support implementation [35]. This toolkit structure has multiple categories, including tools related to definitions, engagement and communication, assessment, planning, training, evaluation, policy and procedure, and finance [35] (see Table 1.) Some tools may meet the purposes of multiple categories; for instance, a tool that is used to evaluate outcomes associated with training may fit into both the “Training” and the “Evaluation” categories.

**Table 1:** Toolkit structure

Category of tools [35]	Purpose of tools in this category [35]
Definitions	Define the intervention
Engagement/communication	Communicate with stakeholders regarding the intervention and increase engagement in the implementation process
Assessment	Determine the current state and identify gaps
Planning	Prepare for implementation
Training	Educate stakeholders
Evaluation	Assess processes or outcomes associated with the implementation
Policy and procedures	Describe the standard processes or rules for operation
Fiscal/funding	Provide information about financial resources or costs

Although investigators used the toolkit structure provided by CalSWEC, the process for developing the toolkit was modified. The CalSWEC resource described a 9-step process that appears to occur linearly and prior to implementation [35]. However, the implementation of an intervention may occur through nonlinear and nonsequential sub-processes occurring simultaneously throughout the organization [36], and leaders and clinicians may find that tools developed prior to implementation may not meet all of their needs. As different strategies are used to support the implementation process over time, new tools may be developed to provide information or guidance. Investigators of this study wanted to modify the toolkit throughout implementation in response to lessons learned about the effectiveness of different implementation strategies. Therefore, the development of the toolkit occurred during three distinct phases throughout the parent study, and the process began by considering the desired clinical outcome and what clinicians needed in order to perform the SBIRT intervention. Phase 1 of toolkit development included preparing for implementation and training site coordinators, phase 2 included completing a readiness assessment at each facility and implementing SBIRT, and phase 3 included reflecting on implementation and refining the toolkit. As tools were created, they were added to the toolkit and disseminated to all investigators and site coordinators. Each site coordinator had access to all tools as the toolkit evolved and selected tools to use or adapt as appropriate to support implementation within their respective facility. Although the tools for this toolkit were developed in three discrete phases, future use of the tools may occur at any point during the implementation process. For example, the tool created to help build a coalition, “Communicating with Stakeholders,” was developed in the final phase of this toolkit development process, but healthcare providers who use this toolkit to implement SBIRT in the future may use this tool early in the implementation process. A summary and timeline of the process for toolkit development are found in Table 2.

**Table 2:** Toolkit development process

Phase	Activities	Dates
Phase 1: Preparation and site coordinator training	- Investigators developed educational and foundational tools	August 2017–December 2017
Phase 2: Readiness assessment and implementation	- Site coordinators developed tools throughout implementation	January 2018–November 2018
Phase 3: Reflection and refinement of the toolkit	- Site coordinators reflected on implementation - Investigators held a focus group to review the toolkit - Site coordinators and investigators refined the toolkit	December 2018 January 2019 February 2019–June 2019

Consistent terminology regarding implementation strategies is an important goal to advance implementation science [12], and the use of consistent terminology and definitions facilitates communication and a common understanding of the purpose of each tool. Because each tool was developed to provide information or guidance related to a specific implementation strategy(s), investigators used the definitions provided by Powell et al. to describe the implementation strategy(s) supported by each tool [12]. Two investigators reviewed each tool to identify up to four relevant implementation strategies supported by the tool. For example, an “Agenda Template” may be used to support the following implementation strategies: (a) create a learning collaborative, (b) capture and share local knowledge, (c) provide ongoing consultation, and (d) organize clinician implementation team meetings.

### Phase 1: preparation and site coordinator training

The first phase of the toolkit development process focused on preparing for the implementation of SBIRT. First, investigators identified the core components of SBIRT required for all hospitals: screening the patient with a validated tool and then providing a brief intervention and referral to treatment when indicated. All other aspects (e.g., who completed the screening, where SBIRT was documented in the medical record, who completed the brief intervention and when this occurred during the hospitalization) were not specified or decided by the investigators. To support successful implementation, investigators planned for the use of several implementation strategies. For example, investigators obtained formal commitments to participate in the study from the chief nursing officer at each facility, the chief nursing officers identified site coordinators to act as champions, investigators prepared site coordinators to lead implementation, and the investigators and nurse leaders worked together to inform local opinion leaders about the implementation of SBIRT.

As investigators and other stakeholders used strategies to prepare for implementation, they created and used tools to provide information or guidance. For instance, the investigators provided information and talking points about the study to chief nursing officers (“Chief Nursing Officer Talking Points”), and then the chief nursing officers provided letters of support to demonstrate their commitment to the study (“Chief Nursing Officer Letter of Support”). Investigators described the site coordinator roles and responsibilities (“Site Coordinator Roles and Responsibilities”), and then the chief nursing officers used this tool to guide their decisions regarding the identification of a site coordinator. Investigators also provided communication tools for use with applicable stakeholders, including email templates about the study and information sheets to share with local opinion leaders (“Email template for Providers” and “Indiana SBIRT Information Sheet”).

To prepare site coordinators for implementation, investigators (including experts in implementation, addiction, and nursing education) developed a curriculum and tools for site coordinator training. The curriculum included foundational knowledge of SBIRT, competency validation, tips for conducting training at the facility level, and strategies for implementation [35]. The education included adjuncts like PowerPoint slides, videos, quizzes, and role-play scenarios. As the investigators prepared for implementation, they reviewed tools used in prior research studies, identified existing tools, or created new tools to support this work.

All site coordinators attended an 8-h training focused on SBIRT foundational knowledge and implementation strategies in the spring of 2018. During the training, site coordinators received all of the relevant tools developed and used in phase 1. A few tools were used by the investigators but not shared with site coordinators (e.g., the “Advisory Council Agenda Template”). Site coordinators unable to attend the training received the toolkit, completed training virtually, and validated competency with a study investigator.

### Phase 2: readiness assessment and implementation

The second phase of the toolkit development process focused on readiness assessment and implementation. During this phase, investigators and site coordinators used several strategies to support implementation, including assessment and identification of barriers and facilitators to implementation; distribution of educational materials; ongoing training, audit and feedback regarding SBIRT fidelity and the implementation process; and adaptation of the intervention. During this phase, site coordinators were able to use and adapt tools that were already available or create new tools to support implementation.

After attending the initial training, each site coordinator used the “Capacity Self-Assessment” tool to complete an assessment for SBIRT implementation at each respective facility. Next, using this self-assessment data, each site coordinator collaborated with investigators and nurse leaders to prepare for implementation. The site coordinators modified the educational tools (e.g., “Substance Abuse Overview”) to conduct training locally. This included role-specific training to assist the key stakeholders in organizing their standard work.

To normalize the new practice, the site coordinators used the engagement and communication tools (e.g., “Indiana SBIRT Information Sheet”) along with face-to-face interactions, to engage key stakeholders, including respiratory therapists, unit registered nurses, social workers, and build commitment by those doing the work. The site coordinators helped the clinical nurses understand the importance of the study, prepared them to invest both time and energy into the study, as well as garnered enthusiasm. Investigators conducted site visits to support site coordinators and observe training and use of the tools.

Although the core evidence-based components of SBIRT were required, the site coordinators had autonomy in how the clinical intervention was embedded in their specific practice setting. On randomly selected days and as needed site coordinators used the “SBIRT Fidelity” tool to assess the degree to which nurses delivered SBIRT as intended. Additionally, site coordinators used the “Implementation Fidelity” tool to assess the degree to which leaders used strategies to support the implementation of SBIRT as intended (e.g., did people receive training on SBIRT, did the site coordinators act upon the gaps identified during the capacity assessment). Site coordinators used information from the SBIRT fidelity and implementation fidelity assessments to provide feedback and coaching as appropriate and modify the use of different strategies to support the implementation process.

Site coordinators also localized strategies based on the organizational context and developed new tools to support implementation. For example, some site coordinators developed a specific process for the provision of SBIRT at his/her facility and created a new tool, “SBIRT Process,” to inform others of this process. This key implementation strategy of adaptation gave the site coordinators the freedom to customize SBIRT to their organizational context. An example of customization was a modification of SBIRT training for respiratory therapy colleagues. In one hospital, respiratory therapy team members were consulted for patients that used tobacco; therefore, an understanding of the SBIRT process was vital. A site coordinator modified the registered nurse training for this stakeholder group by including PowerPoints, videos, and role-playing, and standard work specific to smoking cessation, and then shared this content with other site coordinators. As site coordinators developed new tools to support adaptation, these tools were uploaded to an online collaborative platform shared among investigators and all site coordinators.

To create a learning collaborative and share local knowledge, site coordinators and investigators met monthly (using the “Monthly Meeting Agenda Template”) to discuss progress. Additionally, site coordinators completed the “Monthly Worksheet” tool to monitor the time spent on implementation and associated cost. This was reported during the monthly meetings, along with facilitators and barriers to implementation. Attendees at the monthly meetings also discussed their evaluations of the implementation process and the SBIRT intervention and discussed action items to improve outcomes. Lessons learned and strategies used by the site coordinators were also shared during these monthly discussions. As site coordinators identified challenges with implementation or use of the toolkit, they were able to troubleshoot with their peers within this learning collaborative.

### Phase 3: reflection and refinement of the toolkit

The third phase of the toolkit development process involved reflection on the implementation process and refinement of the final toolkit. Stakeholders reflected individually and as a group considering which tools and implementation strategies were effective, and identified ways to improve the existing toolkit.

After all site coordinators had fully implemented SBIRT at each facility, investigators sent a questionnaire to the site coordinator at each hospital. The purpose of the questionnaire was to stimulate site coordinator reflection on strategies and tools that helped support the implementation of SBIRT. Questions relevant to toolkit development included, “What factors were most helpful in the implementation of SBIRT?” and “What barriers to implementation did you encounter?” Two investigators independently reviewed the responses and then met in person to identify themes to share with a focus group.

To further reflect on the toolkit, investigators held a focus group to discuss strategies to support the implementation of a clinical intervention and determine if the tools included in the SBIRT toolkit were comprehensive. Four members of the research team led the focus group, and attendees included three site coordinators who had been actively engaged in SBIRT implementation throughout the parent study and five stakeholders not previously involved in the SBIRT implementation study (a clinical nurse specialist, a safety/quality consultant, an associate chief nursing officer, and two employees from the Indiana State Department of Health). The additional stakeholders were selected because they had expertise in implementation of clinical interventions in healthcare settings, behavioral healthcare, or substance use disorders. The focus group included an interactive group activity to identify best practices for implementation. Participants were asked to “Reflect on a time when you implemented a practice change with others and you are proud of what you accomplished. What worked well?” Participants reflected silently, and then worked in smaller groups to share their thoughts and identify the strategies that supported the successful implementation of the intervention. Then, each sub-group presented their findings to the full group while an investigator took notes on a whiteboard visible to all participants. Focus group participants then reviewed a summary of the themes identified during the site coordinator survey. Finally, members of the focus group reviewed the SBIRT Toolkit, the themes from the site coordinator questionnaire, and the notes from the focus group reflection to determine gaps in the current SBIRT toolkit. The focus group made several recommendations for change to improve the toolkit, including the addition of a tool to support training regarding motivational interviewing and the addition of a tool to describe how to identify an implementation champion.

After the focus group, site coordinators self-selected into sub-groups based on the toolkit categories (e.g., engagement and communication, assessment, etc.) to finalize the toolkit. The site coordinators reviewed the toolkit, the themes from the site coordinator survey, and the notes from the focus group to ensure the final toolkit was comprehensive and complete. When the sub-groups identified a need for an additional tool, they created the tool at that time (e.g., the “How to Identify a Site Coordinator” tool based on feedback from the focus group).

## Results

The final toolkit is comprised of 54 tools that were identified or developed across the three phases. Table 3 provides a toolkit summary, including the relevant section of the toolkit, the name of the tool, the purpose of the tool, the implementation strategy supported by the tool, and the phase of tool development.

**Table 3:** Implementation toolkit contents

Section of toolkit	Tool name	Purpose of the tool	Implementation strategy [12]	Phase during which the tool was developed
Introduction	Introduction to The Toolkit^a^	Provide toolkit introduction including the purpose of implementation and timeframe	Develop a formal implementation blueprint	Phase 1
Definitions	Acronyms and Abbreviations	List acronyms and abbreviations used throughout the toolkit	Develop an implementation glossary	Phase 1
Engagement/communication	Chief Nursing Officer Letter of Support	Obtain a commitment from leaders to participate in the study and mandate the change	Obtain formal commitments, mandate change	Phase 1
	Chief Nursing Officer Talking Points	Provide information and talking points for each chief nursing officer	Obtain formal commitments	
	E-mail Template for Providers	Provide a template site that coordinators can use to share information with local healthcare providers	Inform local opinion leaders; build a coalition	
	Indiana SBIRT Information Sheet	Summarize SBIRT and the outcomes associated with SBIRT use		
	Advisory Council Agenda Template	Provide a template for meetings with the advisory board	Use advisory boards and workgroups	
Assessment	Capacity Self-Assessment^a b^	Assess capacity, local needs, and barriers and facilitators to implementation	Assess for readiness and identify barriers and facilitators; conduct local needs assessment	Phase 1
Planning	Gantt Chart for Site Coordinators^a^	List site coordinator responsibilities and projected study activities timeline	Prepare champions	Phase 1
	Information for Shared Electronic Platform	Create a common resource to assemble and share information among investigators and site coordinators	Centralize technical assistance	
	Site Coordinator Roles and Responsibilities^a^	Describe the study and anticipated site coordinator roles and responsibilities in order to select a site coordinator	Identify champions	
	How to Identify a Site Coordinator	Describe characteristics of site coordinators that may facilitate implementation	Identify champions	Phase 3
	Training Initial Clinical Nurses and Providing Ongoing Training	Provide a worksheet to inform development of a process to train clinical nurses	Conduct ongoing training	
	Communicating with Stakeholders	Provide a worksheet to help site coordinators identify key talking points and plan for ongoing communication with stakeholders	Build a coalition; create new clinical teams	
	Facility Resources for SBIRT Implementation	Reflect and identify a list of supporting resources already available at the facility	Assess for readiness and identify barriers and facilitators	
	Back-up Plan for Site Coordinator	Develop a process to support SBIRT implementation when the site coordinator is not available	Create new clinical teams	
	How to Adapt the Intervention	Identify the core components of the intervention and the components of the intervention that may be adapted	Promote adaptability	
Training	Pre-course Training	Provide a short introduction to SBIRT and the study before site coordinators attend the 8-h training	Develop and distribute educational materials; prepare champions	Phase 1
	Training Agenda	Provide an agenda and objectives of the site coordinator training		
	List of Investigators	Familiarize the site coordinators with the study investigators	Create new clinical teams; prepare champions	
	List of Site Coordinators	Generate a list of names and contact information		
	Study Overview	Provide a brief description of the study that site coordinators may share with stakeholders	Distribute educational materials; use train-the-trainer strategies, prepare champions; make training dynamic	
	Study Timeline	Provide a timeline of all study activities including training and data collection		
	Study Introduction	Provide detailed information about the study		
	Substance Abuse Overview	Teach site coordinators about substance use disorders, including epidemiology, risk factors, and the neurobiology of addiction		
	SBIRT Introduction	Educate site coordinators regarding the evidence supporting SBIRT and the value of implementing SBIRT		
	Screening for Substances	Describe tools used to screen for drug and alcohol use and demonstrate how to screen patients		
	Motivational Interviewing	Describe stages of change and the principles of motivational interviewing; explain the goals and components of brief interventions		
	Stages of Change Exercise	Discuss different stages of change and demonstrate potential responses		
	System Issues and Implementation	Guide discussion regarding implementation at each facility		
	Additional SBIRT Resources	List websites, online videos, and other resources that provide additional information about substance abuse and SBIRT		
	Documentation Process	Describe a standard process to document SBIRT in the electronic medical record and provide examples of documentation		
	Protocol for Missed Site Coordinator Training	Describe the steps to complete training if the site coordinator was unable to attend the 8-h training session		
	Patient Education Brochure- Ordering Info	Provide information to order patient education brochures		
	Brief intervention Cheat Sheet	Provide a one-page guide to motivational interviewing	Distribute educational materials; capture and share local knowledge; Tailor strategies	Phase 2
	Brief Intervention Guide Using the 5 A’s	Provide a guide to intervening using (Ask, Assess, Advice, Assist, and Arrange)		
	Documentation Badge Reference	Provide a quick guide to documentation that can be worn with the nurse’s identification badge		
	REWARD Smoking Cessation	Provide patient education using the REWARD acronym with benefits of quitting smoking		
	Education Express	Notify direct care nurses of the new SBIRT process and go-live date		
	Motivational Interviewing Article	Provide additional details about motivational interviewing	Conduct ongoing training, distribute educational materials	Phase 3
Evaluation of training	SBIRT Knowledge Assessment^b^	Assess knowledge of SBIRT after the completion of training	Distribute educational materials; use train-the-trainer strategies, prepare champions	Phase 1
	Brief Intervention Adherence and Competence Scale^b^	Demonstrate competency providing a brief intervention using motivational interviewing techniques	Distribute educational materials; capture and share local knowledge	
Policy and procedure for training (non-localized)	SBIRT Documentation Form	Provide a paper document for completion of the screening tools and brief intervention (note: SBIRT is not located in the electronic medical record at this time)	Distribute educational materials; use train-the-trainer strategies, prepare champions	Phase 1
	SBIRT Flowchart	Provide a step-by-step flowchart of the components of SBIRT		
Evaluation of implementation and intervention fidelity	Chart Data Abstraction Tool	Collect data from patient’s electronic medical records regarding screening, brief intervention, and referral to treatment	Audit and provide feedback	Phase 1
	Implementation Fidelity^b^	Assess fidelity to the implementation process		
	Monthly Meeting Agenda Template	Provide a template for the monthly site coordinator/investigator meeting	Create a learning collaborative; capture and share local knowledge; ongoing consultation; organize clinician implementation team meetings	
	Monthly Worksheet**	Collect information regarding site coordinator time spent implementing SBIRT, barriers/facilitators to change, and lessons learned from implementation	Identify barriers and facilitators; capture and share local knowledge	
	SBIRT Fidelity^b^	Assess fidelity to the SBIRT process	Develop and implement tools for quality monitoring	
Policy and procedure (localized)	SAMSHA-Approved Providers	List treatment centers (including the address and phone number) throughout the state	Tailor strategies	Phase 1
	SBIRT Process 2 (localized to a specific facility)	Describe the process for completing SBIRT, including the people responsible and hand-off between providers	Promote adaptability	Phase 2
	Documentation Form with Barcode (multiple versions localized to different facilities)	Provide a document for completion of the screening tools and brief intervention (note that screening tools are not located in the electronic medical record at this time)	Promote adaptability; change record systems; tailor strategies	
	SBIRT Process (multiple versions localized to different facilities)	Describe the process for completing SBIRT, including the people responsible and hand-off between providers	Promote adaptability; tailor strategies	
Fiscal and funding	SBIRT Reimbursement Resources	List billing codes that have been used for SBIRT	Make billing easier	Phase 2

^a^Modified in phase 3

^b^Also used in prior SBIRT study [32]

During phase 1, study investigators identified 38 tools (Table 3). Six of the tools were used in a previous study [37], and 32 tools were developed or selected by investigators for this study. Most of the tools from the first phase (*n* = 20) relate to training the site coordinators and preparing them to train clinical nurses and champion the implementation process at their facility. Five tools support evaluation of the implementation process and SBIRT fidelity using strategies such as auditing and providing feedback, creating a learning collaborative, sharing local knowledge, and ongoing consultation. Five tools facilitate engagement and communication with stakeholders by informing stakeholders, building a coalition, obtaining formal commitments to participate in the study, and gathering feedback from an advisory board. Three tools guide planning for implementation by identifying and preparing champions as well as identifying a place to centralize technical assistance. Additional resources created in phase 1 included strategies to introduce the toolkit, define terms, assess for readiness, and describe policies and procedures.

During phase 2, site coordinators developed nine tools (Table 3). The majority of the tools (*n* = 5) included information to train clinical nurses; site coordinators identified gaps in knowledge and created tools to address the gaps and share this knowledge with others. Three tools supported adapting the intervention to fit the organizational context. These tools described the policy, procedure, or process for SBIRT implementation at a specific facility. One tool listed potential reimbursement options for SBIRT. To train clinical nurses at each facility, several site coordinators created condensed versions of the training materials developed in phase 1. Because these abbreviated versions of training material did not include new content, they were not included in the final toolkit.

During phase 3, investigators and site coordinators created seven new tools and modified four existing tools (Table 3). Six tools were created to help plan for implementation by identifying champions, creating new teams, building a coalition, identifying facilitators to implementation, promoting adaptability, and conducting ongoing training. While these activities occurred throughout implementation, site coordinators stated in the questionnaires and during the focus group that additional tools to support these strategies would have been helpful. One tool was selected to support ongoing training regarding the brief intervention. All four of the existing tools modified in phase 3 had minor changes to enhance clarity.

## Discussion

Several strategies can be used to facilitate the implementation of evidence-based interventions to decrease the gap between evidence and practice and improve healthcare delivery and outcomes. The toolkit described in this paper provides a compilation of tools to provide information or guidance to support implementation of SBIRT in acute care settings. The toolkit structure and development process can be used for a variety of interventions and settings. For example, leaders implementing a clinical intervention in home care, emergency departments, or community settings may follow the same 3-step process to develop a toolkit: develop educational and foundation tools for the intervention, create new tools throughout implementation, and then reflect and refine the toolkit. This approach provides foundation to begin implementation and allows for stakeholder input and feedback to promote engagement and ownership of the practice which may enhance sustainment of the clinical intervention.

Taking time to understand the various needs and available resources within hospitals of different sizes throughout the state of Indiana enhanced the investigator and site coordinator collective problem solving among sites. Investigators of the parent study [34] encouraged the adaptation of the intervention to the organizational context. This flexibility supported additional tools and resources to emerge organically and ultimately contributing to the final toolkit content. Engagement of end-users and the use of an iterative process was essential to refine the toolkit. A limitation of this toolkit is that all of the stakeholders worked within one healthcare system, and tools may not be applicable in different settings. For example, the “Documentation Process” is specific to the electronic medical record used in this healthcare system.

Similar to the development of the SBIRT toolkit, consensus building was a popular method for finalizing content for evidence-based toolkits [18, 20]. Ultimately, the perceived value expressed through clinician engagement on each component of the toolkit structure was essential to the development of the final toolkit. Considering that clinicians were the developers of several of the components of the final toolkit, the tools support implementation strategies used by clinicians in real-world settings. Because of their active role in developing the tools, site coordinators could make suggestions and propose strategies based on their context and identified needs. Allowing flexibility to adapt and refine innovations can improve the implementation process [38], and implementation toolkits grounded in consensus have the potential to enhance intervention adoption and sustainment.

There are several limitations to this case study. First, this case study describes the development and use of a toolkit, but a case study method lacks scientific rigor and may limit the generalizability of the results to broader populations. Second, the structure of the toolkit and the process for toolkit development are described, but it would be challenging to replicate the exact methods or achieve the same results. Third, the investigators were invested in the success of SBIRT implementation, which could introduce bias during data collection and data analysis.

By continually monitoring implementation and developing tools throughout the implementation process, the components of the toolkit provide resources throughout adoption (deciding to use), implementation (using), and sustainment (continuing to use) of SBIRT. The organizational elements that influence the use of evidence-based interventions, leadership, culture, resources, communication, evaluation methods, and champions/site coordinators [39] were incorporated into the toolkit to support sustainability specific to the SBIRT intervention. Planning for sustainability should be part of the implementation plan and includes establishing flexibility in adapting the evidence-based intervention to new or evolving populations, policies, evidence, and other contextual factors [40].

## Conclusion

Toolkits may be used to support the implementation of evidence-based interventions. One intervention, SBIRT, has direct health benefits to patients through recognition of substance use health risks and connection to appropriate treatment when indicated. This case report describes a toolkit developed by investigators and nurses in clinical settings to facilitate the implementation of SBIRT. Investigators identified core components of the clinical intervention, and then site coordinators received training and tools to prepare for implementation. Training includes the components of the clinical intervention that had to remain the same at all hospitals and the elements that could be adapted to fit the context of each hospital. As site coordinators lead implementation at each hospital and adapted implementation, new tools were developed to provide information and guidance to stakeholders. Finally, investigators and site coordinators reflected on the implementation process and refined the toolkit. Results from this case report can support health systems to consider adaptation of evidence-based interventions to their local context, with the potential to improve care delivery. Incorporating evidence-based clinical interventions into practice is challenging, and the creation of a toolkit can provide resources to inform and guide implementation. Learning nuances of how to implement within the clinician workflow successfully can provide direction for future efforts to build an infrastructure for the management and sustainment of evidence-based interventions.

## Data Availability

n/a

## Abbreviations

SBIRT: Screening, Brief Intervention, Referral to Treatment
